# Heterotopic cervical pregnancy: Case report and literature review

**DOI:** 10.1016/j.radcr.2025.02.081

**Published:** 2025-03-20

**Authors:** Ghazaleh Salehabadi, Nedasadat Rezaei, Ayda Roostaee, Nooshin Eshraghi, Zeinab Safarpour Lima

**Affiliations:** a Department of Radiology, School of Medicine, Iran University of Medical Sciences, Tehran, Iran; b Department of Obstetrics and Gynecology, School of Medicine, Shahid Akbar-Abadi Hospital, Iran University of Medical Sciences, Tehran, Iran

**Keywords:** Case report, Heterotopic pregnancy, *In-vitro* fertilization, Transvaginal ultrasonography

## Abstract

Heterotopic cervical ectopic pregnancy (CEP) is a rare form of ectopic pregnancy, that involves implantation of gestational sac in both the cervical canal and uterine cavity. This condition poses high risks of severe complications like lethal vaginal bleeding and abortion. Transvaginal ultrasonography is the primary diagnostic tool. Risk factors may vary from prior cesarean delivery to the utilization of Assisted Reproductive Technologies (ART). Despite the research conducted on this condition, the best treatment strategy has not been established. This study discusses a rare case of cervical heterotopic pregnancy, emphasizing early diagnosis. Our case underwent in-vitro fertilization (IVF). We used potassium chloride (KCL) and methotrexate (MTX) as treatment. At first, the cervical gestational sac was expelled but unfortunately, later, the intrauterine gestational sac was also aborted. We also reviewed 25 cases (1980-2024) which demonstrated the importance of early diagnosis of heterotopic cervical pregnancy and that most cases are likely to have positive outcomes.

## Introduction

Ectopic pregnancy, characterized by the implantation of a fertilized ovum outside the uterine cavity or in an inappropriate place in the uterus such as interstetium, cornual part or cervix, affects approximately 1%-2% of pregnancies in the United States. However, due to the potential for early diagnosis and treatment in outpatient settings, the true prevalence may be higher [1,2]. Patients with EP may experience nonspecific symptoms, including lower abdominal pain and vaginal bleeding. These symptoms can also represent other conditions, such as appendicitis, urinary tract infections, early pregnancy loss, or pelvic trauma [3].

Cervical ectopic pregnancy (CEP) is a rare form of ectopic pregnancy, occurring in approximately 1 in 8,600 to 12,400 pregnancies [4]. It is represented by the implantation of the fertilized ovum within the cervical canal, below the internal OS [5]. Spontaneous heterotopic pregnancy is also a rare condition characterized by the simultaneous occurrence of both intrauterine and extrauterine gestations [6]. The most common type of heterotopic pregnancy is intrauterine pregnancy combined with tubal pregnancy, while intrauterine pregnancy combined with cervical pregnancy is very rare. CEP is a serious condition associated with significant morbidity and mortality. Delayed diagnosis and treatment can lead to severe complications, including severe bleeding requiring hysterectomy [5]. Previous cesarean deliveries, dilation and curettage procedures, history of pelvic inflammatory disease, cigarette smoking, fallopian tube surgery, prior ectopic pregnancy, infertility, and assisted reproductive technologies (ART) are risk factors for cervical implantation [7,8]. Despite advancements in ultrasound technology, early diagnosis of heterotopic pregnancy remains challenging due to its often-asymptomatic nature in the initial stages.

We present a case of rare cervical heterotopic ectopic pregnancy, concentrating more on the significance of early diagnosis for optimal maternal and fetal management. We also present a literature review of 25 cases, reported from 1980 to 2024.s

## Case presentation

A 32-year-old primigravida female with a history of endometriosis, who got pregnant via in vitro fertilization (IVF), presented for routine check-ups at Shahid Akbarabadi Hospital of Tehran. A transvaginal ultrasound of the uterus and adnexa revealed a gestational sac (GS) in the proximal uterine cavity containing a yolk sac but no fetal pole (FP). Based on a mean sac diameter (MSD) of 13 mm, the gestational age was estimated to be 6 weeks and 1 day. An intramural to submucosal fibroid measuring 29 × 19 mm was also noted, exerting mild pressure on the posterior aspect of the gestational sac in the uterine body.

**Fig. 1:**
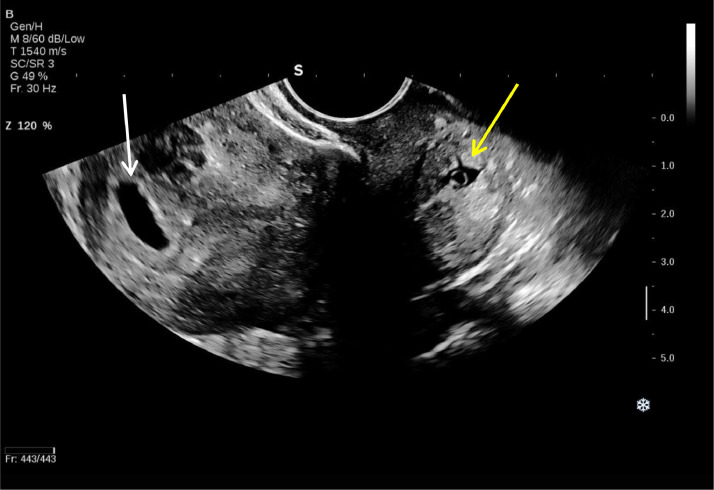
Transvaginal ultrasound revealed a GS in the proximal uterine cavity (white arrow) and a GS in the posterior wall of distal cervix (yellow arrow) suggestive of cervical heterotopic pregnancy.

**Fig. 2:**
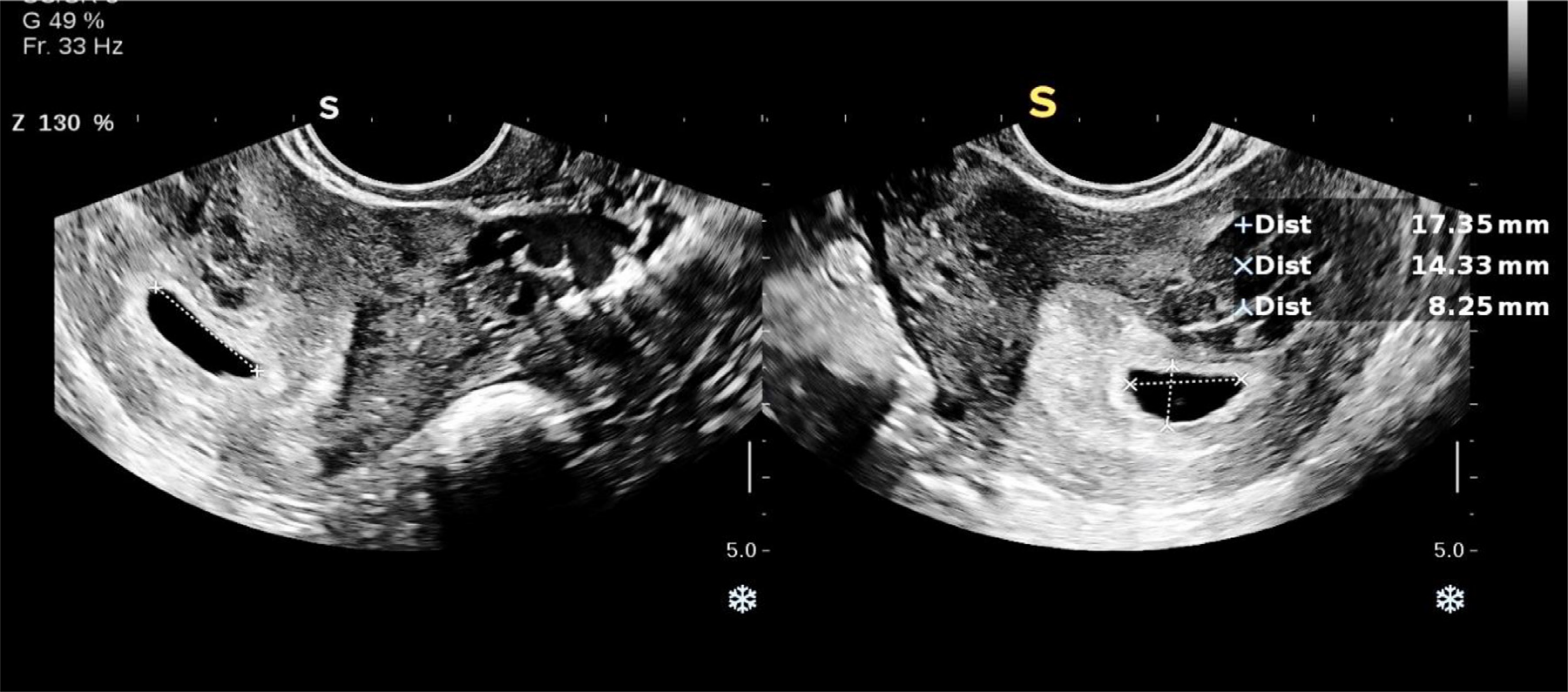
Transvaginal ultrasound revealed a gestational sac in the proximal uterine cavity containing a yolk sac but no fetal pole (FP).

**Fig. 3:**
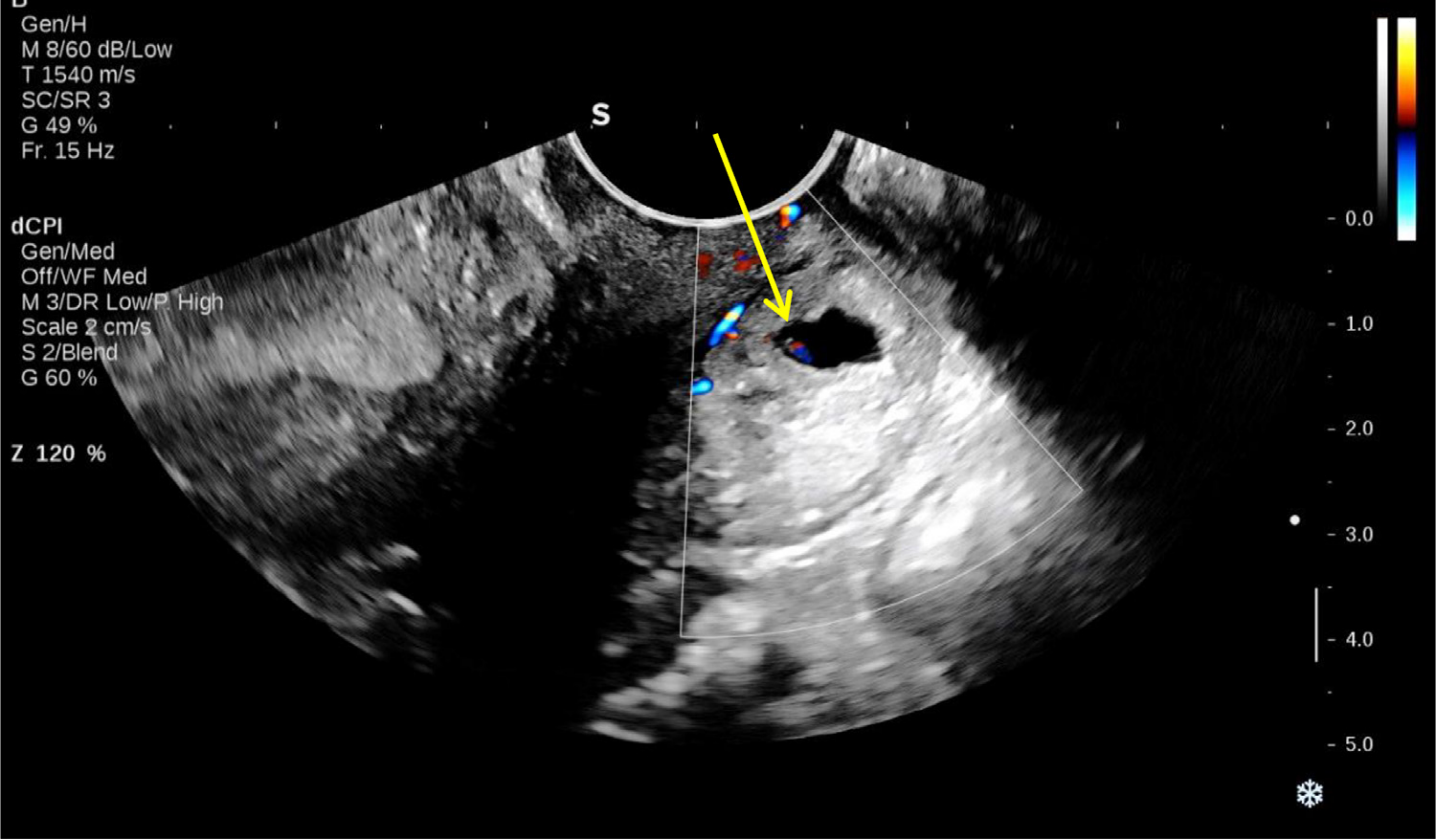
Transvaginal ultrasound revealed GS containing a viable embryo with a CRL of 3 mm and gestational age of 5 weeks and 6 days, in the posterior wall of distal cervix (yellow arrow).

A smaller gestational sac containing a viable embryo with a crown-rump length (CRL) of 3 mm, corresponding to a gestational age of 5 weeks and 6 days, was visualized in the posterior distal cervix, indicative of a cervical ectopic pregnancy (CEP). The distance from the cervical gestational sac to the external OS, considering the decidual reaction, was approximately 5 mm. Myometrial thickness was 9 mm anteriorly and 4 mm posteriorly, excluding the decidual reaction. Based on the findings, a heterotopic pregnancy was diagnosed, comprising an intrauterine pregnancy without a visible embryo and an ectopic gestational sac in the cervix (Fig. 1, Fig. 2, Fig. 3).

**Fig. 4:**
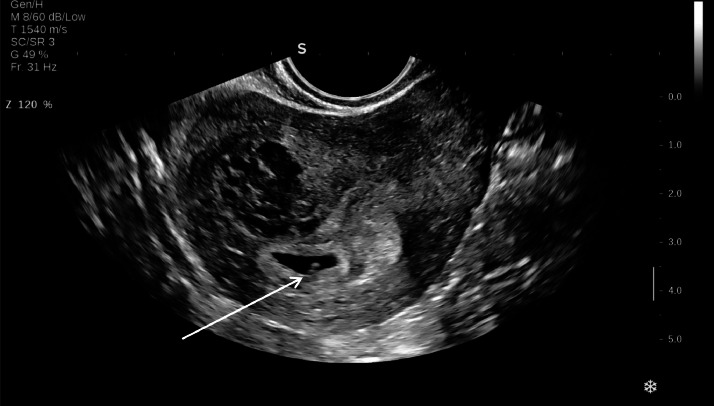
Transvaginal ultrasound showed an intrauterine pregnancy, containing a FP with FHR (white arrow).

**Fig. 5:**
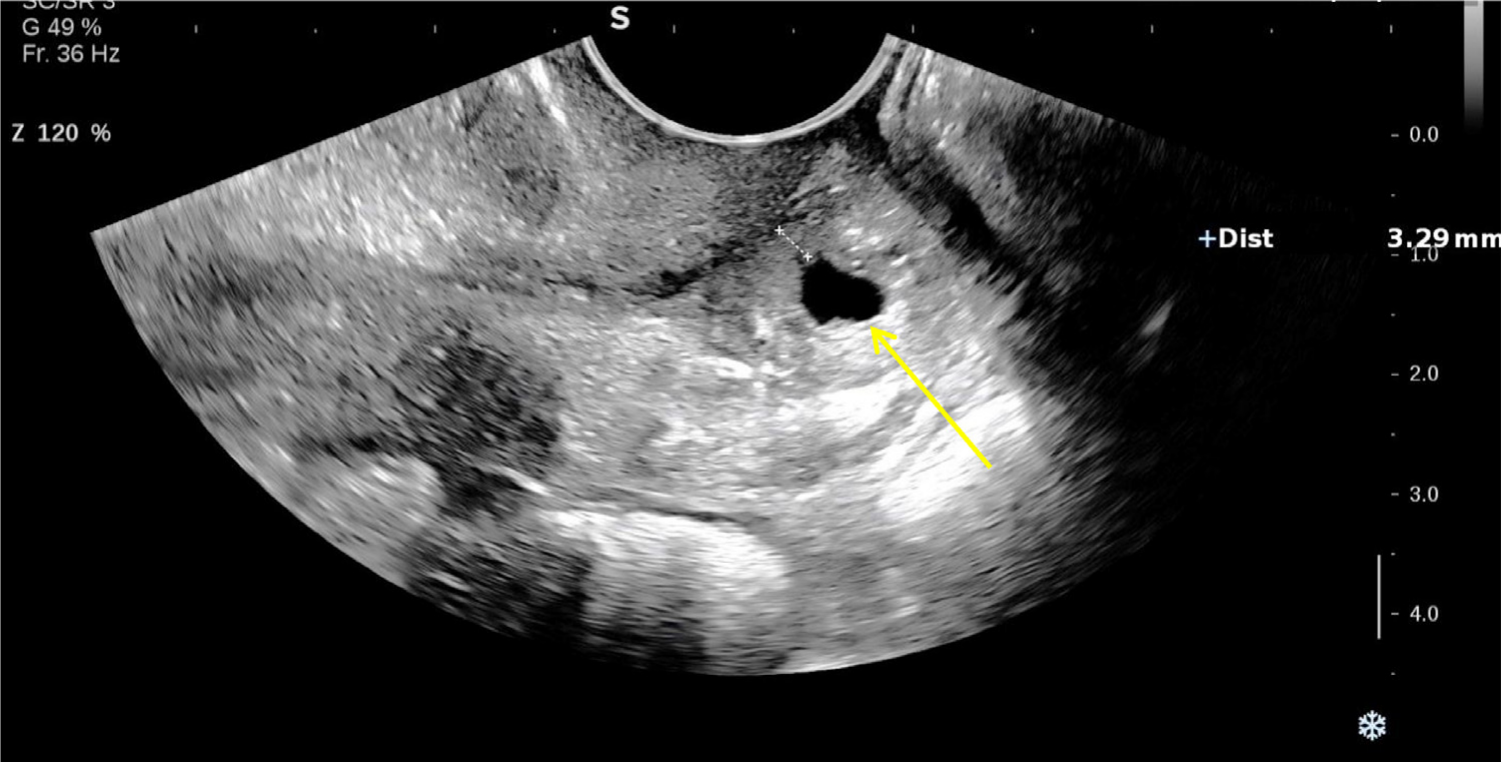
Transvaginal ultrasound showed the cervical gestational sac measuring 15 × 14 mm and no cardiac activity was detected in the FP anymore suggestive of embryo demise (yellow arrow).

Six days after the initial diagnosis, intracervical injections of potassium chloride (KCl) and methotrexate (MTX) were administered for the cervical ectopic pregnancy. A follow-up ultrasound at the same day showed a subchorionic hematoma measuring 16 × 18 mm adjacent to the intrauterine gestational sac. The intrauterine pregnancy was located appropriately, containing a FP with fetal heart rate (FHR) = 138 beats/min (bpm) and CRL corresponding to 6 weeks and 2 days (Fig. 4). The cervical gestational sac measured 15 × 14 mm, and no cardiac activity was detected in the FP anymore suggestive of embryo demise (Fig. 5).

On the next day, the patient experienced pain, and the cervical gestational sac was expelled.

In a color doppler ultrasound performed that day, the intrauterine GS contained a fetal pole with FHR of 126 bpm. Subchorionic hematoma measuring 19 × 26 mm, encompassing approximately 5% of the sac circumference, was observed.

The day after the abortion of cervical GS, the patient experienced pain and went through heavy bleeding, the intrauterine GS was also aborted. Misoprostol was prescribed to facilitate the evacuation of retained products of conception, blood and clots.

In a follow-up color Doppler ultrasound performed the day after the abortion of intrauterine GS, the uterus measured 90×60×46 mm. No distinct gestational sac was visible within the endometrial cavity. Two hyperechoic heterogeneous areas measuring 23×16 mm and 32×19 mm with internal vascularity lower than the adjacent myometrium and, suggestive of retained products of conception (RPOC) grade I, were noted in the cervical canal and the proximal part of the endometrial cavity respectively.

The patient underwent dilation and curettage (D&C), and Samples of uterine contents, preserved in 2 formalin containers, were sent to the pathology laboratory for microscopic and macroscopic evaluation. One container containing expelled uterine contents was macroscopically described as multiple pieces of creamy-brown soft tissue with a total size of 4 × 3 × 1.5 cm. Half of this volume was transferred to a basket, and 50% of the sample was passed. The second container contained curettage material, similarly, described macroscopically as creamy-brown soft tissue, with a total size of 3 × 2 × 1 cm. Half of this sample was transferred to a basket, and 70% of the sample was passed. Microscopic examination confirmed the presence of products of conception (POC) in both samples.

## Discussion

In this study, we presented the diagnostic and therapeutic process of a case of heterotopic cervical pregnancy (CP) after IVF. The diagnosis was made using transvaginal ultrasound and achieved in a timely manner. Fortunately, the ectopic pregnancy (EP) was successfully expelled, and maternal health was preserved. However, despite all efforts, we were unable to sustain the intrauterine pregnancy. Additionally, we conducted a literature review focusing on the diagnosis and management of CP to provide further context and insight.

The first documented ultrasound findings of cervical pregnancy were described by Raski in 1978 [9]. The prevalence of cervical pregnancy (CP) is exceedingly rare, accounting for less than 1% of all EPs [10]. Studies indicate that a significant proportion of EP patients, ranging from 50% to 90%, have previously undergone uterine curettage [11]. Additional potential risk factors have been recognized, such as endometrial inflammation, disruptions in the intrauterine environment, the use of intrauterine devices (IUDs), structural uterine anomalies, the presence of uterine fibroids, congenital fetal defects, and the application of assisted reproductive technologies (ART) [12]. Untreated cervical pregnancy has significant risks, including severe bleeding, the potential need for hysterectomy, or even death. These dangers become more notable when a cervical pregnancy coexists with an intrauterine pregnancy, necessitating careful consideration of preserving the intrauterine pregnancy during the treatment process [13]. The rise in ectopic pregnancies associated with IVF procedures remains inadequately understood. The transfer of multiple embryos during IVF could contribute to a higher likelihood of various implantations. Additionally, the elevated hormone levels induced during ovarian stimulation might negatively impact endometrial receptivity, potentially influencing implantation outcomes [14]. Also, factors such as infertility, cervical injury, and embryo reflux resulting from embryo transfer (ET) may contribute to an increased risk [15]. In most women with heterotopic pregnancy located in the cervical region of the uterus, symptoms typically present as vaginal bleeding [16]. Diagnosis is often achieved through transvaginal ultrasonography. In some cases, pelvic Magnetic Resonance Imaging (MRI) examination is also utilized for diagnostic purposes [17,18]. Most cases of this type of pregnancy are identified and diagnosed between weeks 5 and 8 (approximately 70%). Diagnosis occurs in 20% of cases between weeks 9 and 10, and in 10% of cases after week 11 [19]. The choice of treatment method for heterotopic cervical pregnancy depends on various factors, including the gestational age, the mode of conception, the fetal cardiac status, and the mother's preference regarding the preservation or termination of the pregnancy [20]. Treatment options can be surgical or conservative. Surgical methods include procedures such as aspiration, curettage, hysteroscopy, or uterine artery embolization. Conservative approaches involve the local or systemic injection of agents like methotrexate (MTX), potassium chloride (KCl), high-concentration sodium chloride (NaCl), or glucose. If the mother requests to preserve the pregnancy, it is essential to carefully consider the potential adverse effects of MTX and uterine artery embolization on the fetus and the pregnancy. This decision-making process requires a multidisciplinary approach to ensure maternal safety and the best possible outcome for the pregnancy [13].

To analyze the symptoms, different forms of treatment, and relatable outcomes of heterotopic cervical pregnancy we reviewed 25 relevant case reports from 1980 to 2024 (Table 1).

**Table 1:** Overview of published heterotopic cervical pregnancy case reports.

Year	Authors	Maternal Age	Gravida	Para	Pregnancy way	Medical history	Symptoms	Embryos implanted	Embryo morphology	Time to first detect	Treatment	Pregnancy outcome
1980	Giorgio et al. [21]	30	Not mentioned	Not mentioned	Spontaneous	Not mentioned	Painless vaginal bleeding	One in the cervix	-	9 wk pregnancy	Suction aspiration curettage and suture	Intentional abortion
1994	Centini et al. [22]	36	1	1	Spontaneous	Cesarean section	diffuse pains in the lower pelvis	One in the cervix	-	6 wk+ 2 d	Aspiration, curettage, and methotrexate	Intentional abortion
2001	Chen at al. [15]	35	1	0	ICSI	Bilateral salpingectomy, polypectomy	Mild vaginal bleeding	One intrauterine and one in the cervix	Cleavage embryo	25 d after ET	Aspiration, local injection of KCl, cerclage (10 wk)	Live birth through cesarean section at 38 wk
2003	Cepni I et al. [23]	27	Not mentioned	0	Spontaneous	Unremarkable	vaginal bleeding, lower abdominal pain, and nausea	one in the cervix	-	7 wk	Aspiration and systematic methotrexate	Intentional abortion
2003	Porpora et al. [24]	29	4	1	Spontaneous	Left salpingectomy	Asymptomatic	One intrauterine and one in the cervix	-	6 wk	Aspiration	Abortion (1 day after aspiration)
2006	Ujvari et al. [25]	27	0	0	IVF-ET	bilateral occlusion of uterine tubes	Asymptomatic	Two intrauterine and one in the cervix	Cleavage embryo	4 wk after ET	Aspiration	Two live births through cesarean section at 29 wk
2007	Cho et al. [26]	35	Not mentioned	Not mentioned	IVF-ET	Not mentioned	Asymptomatic	One intrauterine and one in the cervix	Not mention	7 wk	Aspiration	Delivery of a healthy infant at 35 wk
2007	Prorocic and Vasiljevic [27]	31	Not mentioned	Not mentioned	IVF-ET	Bilateral salpingectomy	Vaginal bleeding	Two in the uterine and one in the external cervical ostium	Not mentioned	6 wk	Aspiration and local injection of hypertonic solution of sodium chloride	Ongoing pregnancy
2007	Suzuki et al. [28]	35	0	0	IVF-ET	Unremarkable	Asymptomatic	Two intrauterine and one in the cervix	Not mention	24 d after ET	Aspiration and local injection of 33% glucose solution	Two live births through cesarean section at 34 wk
2009	Shah et al. [29]	34	4	2	IVF/ICSI	Myomectomy, cesarean section, curettage	Asymptomatic/ painless vaginal bleeding after aspiration	One intrauterine and one in the cervix	Cleavage Embryo	34 d after ET	Aspiration/ internal iliac artery balloon catheters placement before delivery	Live birth through cesarean section at 37 wk
2009	Kim et al. [30]	30	1	0	Spontaneous	Not mentioned	Asymptomatic/ bleeding during aspiration	one intrauterine and one in the cervix	-	8 wk	Aspiration and Foley catheter insertion	Live birth through cesarean section at 37 wk
2010	Faschingbauer et al. [31]	25	Primigravida	0	clomiphene citrate/ spontaneous	Unremarkable	vaginal bleeding	One intrauterine and one in the cervix	-	9 wk	Aspiration and cerclage	Live birth through vaginal delivery at 39 wk+3 d
2015	Tsakos et al. [32]	41	2	0	IVF-ET	History of ectopic cervical pregnancy	Asymptomatic	One intrauterine and one in the cervix	Blastocyst‐stage embryo	7 wk+ 5d	Aspiration, Foley catheter insertion, cerclage	Live birth through cesarean section at 38 wk
2019	Drezett et al. [33]	36	0	0	IVF-ET	Hyperprolactinemia	Vaginal bleeding	One in the cervix	Cleavage embryo	36 d after ET	Aspiration under laparoscopy	Intentional abortion
2021	Mu et al. [34]	34	5	2	Not mentioned	Surgery for a left eye vascular tumor	Amenorrhea and irregular vaginal bleeding	One intrauterine and one endocervical gestational sac	Not mentioned	7+ wk	Aspiration, uterine artery embolization	Total abortion
2021	Koutras et al. [35]	30	3	2	Spontaneous	Unremarkable	Lower abdomen pain and vaginal bleeding	One intrauterine and one in the cervix	Not mentioned	7W + 4d	Mifepristone, synthetic steroid, misoprostol, prostaglandin E1, curettage, Foley balloon	Total abortion
2022	Fan et al. [20]	29	Not mentioned	0	IVF-ET	Not mentioned	Vaginal bleeding, abdominal pain, dizziness	One intrauterine and one endocervical gestational sac	Cleavage	42 d after ET	CP aspiration under ultrasound guidance, tranexamic acid gauze, 800 mL blood transfusion	C-section delivery at 39 wk
2022	Fan et al. [20]	27	1	1	ICSI-ET	Right salpingectomy, ART treatment	Mild vaginal bleeding	One intrauterine and one endocervical gestational sac	Cleavage	36 d after ET	CP aspiration under ultrasound guidance, tranexamic acid gauze	Vaginal delivery at 27 wk
2022	Tan et al. [36]	29	Not mentioned	Not mentioned	IVF-ET	Not mentioned	Not mentioned	One intrauterine and one endocervical gestational sac	D5 blastocysts	20 d after ET	Aspiration of cervical gestational sac under vaginal ultrasound guidance	Delivery at 20 wk and 6 d
2022	Tan et al. [36]	30	Not mentioned	Not mentioned	IVF-ET	Not mentioned	Asymptomatic	One intrauterine and one endocervical gestational sac	D3 blastocysts	24 d after ET	Aspiration of cervical gestational sac under vaginal ultrasound guidance	Successful delivery
2022	Sheng et al. [13]	31	2	0	IVF-ET	Left salpingectomy, lost left fallopian tube	Asymptomatic	One intrauterine and one endocervical gestational sac	Not mentioned	33 d after ET	Ultrasound-guided hysteroscopic surgery, Foley catheter, iodine gauze	C-section delivery at 38 wk
2023	Valdés-Martínez et al. [37]	35	Not mentioned	0	IVF-ET	History of infertility and C-section	Mild transvaginal bleeding	One intrauterine and one cervical gestational sac	Not mentioned	6.2 wk	Puncture of cervical gestational sac with ultrasound guidance, aspiration of a milliliter of amniotic fluid, 100 mg of MTX	Live birth through cesarean section at 38.4 wk
2023	Valdés-Martínez et al. [37]	28	1	1	Not mentioned	Previous C-section	Moderate transvaginal bleeding	Only one cervical gestational sac	Not mentioned	7.1 wk	Puncture of cervical gestational sac with ultrasound guidance, aspiration of a milliliter of amniotic fluid, 100 mg of MTX	Intentional abortion
2023	Valdés-Martínez et al. [37]	40	Not mentioned	0	IVF-ET	2-yearof infertility	Asymptomatic	Only one cervical gestational sac	Not mentioned	2 wk	Puncture of cervical gestational sac with ultrasound guidance, aspiration of a milliliter of amniotic fluid, 100 mg of MTX	Intentional abortion
2024	Alexakis et al. [38]	33	2	0	IVF-ET	Not mentioned	Excessive nausea and vomiting	One intrauterine and one endocervical gestational sac	Blastocyst	6 wk	Ultrasound-guided KCL injection in to cervical sac	Delivery at 36 wk and 6 d

Among the cases where the way of pregnancy was specified (*n* = 23), the majority (56.52%) conceived through IVF-ET (*n* = 13). 30.43% of patients achieved pregnancy spontaneously (*n* = 7). Additionally, 2 cases were conceived via Intracytoplasmic Sperm Injection (ICSI)-ET, and one case through IVF/ICSI. The maternal age at diagnosis varied between 25 and 41 years, with a mean of 31.88 years. 39.13% of individuals were diagnosed with their condition within 20-42 days after ET (*n* = 9). All of these 9 pregnancies were achieved through IVF-ET, ICSI-ET, and IVF/ICSI. The rest of the cases were diagnosed between 2 weeks and 9 weeks of pregnancy. 21.73% of cases had a history of salpingectomy (*n* = 5). 17.39% of the cases had a history of C-section delivery (*n* = 4). As for clinical presentation, 43.48% were asymptomatic (*n* = 10). The most common symptom was vaginal bleeding (*n* = 12; 52.17%). Four cases experienced pelvic or abdominal pain. Other common symptoms were nausea, dizziness, and vomiting. Six cases had only one cervical gestational sac with no intrauterine gestational sac; therefore, all of these cases underwent intentional abortion. The rest tried their best to preserve the intrauterine pregnancy. Fourteen of them achieved term deliveries, while one was still pregnant at the time of reporting, and another experienced a miscarriage one day after aspiration. By analyzing the treatment methods in Table 1, we can see that 73.9% of patients underwent aspiration (*n* = 17). Of these patients, only 26.09% of them underwent aspiration alone. The others were treated with additional options like curettage (*n* = 3), cerclage (*n* = 3), MTX (*n* = 2), and foley catheter insertion (*n* = 2). KCL, hypertonic NaCl solution and glucose were each used individually in 1 case alongside aspiration.

## Conclusion

In conclusion, heterotopic pregnancy remains a rare but life-threatening condition. Early diagnosis and prompt management are crucial to optimize maternal and fetal outcomes. Advanced imaging techniques, such as ultrasound and MRI, play an important role in the diagnosis of this condition. While conservative management may be considered in selected cases, surgical intervention is often necessary to remove the ectopic pregnancy and preserve the intrauterine pregnancy. Based on our study the majority of cervical heterotopic pregnancy cases tend to have positive outcomes, including successful live births and preservation of maternal fertility. However, this perspective may be overly optimistic, as adverse outcomes are less likely to be documented and published. Future research is needed to explore further the risk factors, pathophysiology, and optimal management strategies for heterotopic pregnancy.

## Author contributions

Conceptualization: G.S, N.R, N.E, A.R, Z.S.L; Writing—original draft preparation: G.S, N.R, A.R, Z.S.L; Writing—review and editing: G.S, N.R, Z.S.L.

## Patient consent

Signed consent was taken from the patients which can be made available upon reasonable request from the lead author.
